# Editorial: Red Blood Cell Vascular Adhesion and Deformability

**DOI:** 10.3389/fphys.2020.00657

**Published:** 2020-06-24

**Authors:** Helene Guizouarn, Gregory Barshtein

**Affiliations:** ^1^CNRS laboratory Institut Biology Valrose, Nice, France; ^2^Department of Biochemistry, The Faculty of Medicine, The Hebrew University of Jerusalem, Jerusalem, Israel

**Keywords:** RBC—red blood cell, RBC deformability, RBC deformation and aggregation, RBC adhesion, erythrocyte

In the present Research Topic, the red blood cell (RBC) flow-affecting properties are examined from different angles of view, revealing the complexity of these characteristics and their role in ensuring proper blood circulation.

The primary role of RBCs is the transport of respiratory gases, oxygen, and CO_2_, which is carried out mainly in the blood capillaries. The ability of blood to circulate is largely determined by the flow-affecting properties of RBCs (Jani et al.). Under the flow, RBCs export vasoactive mediators in response to deformation and other physiological or pathological stimuli (McMahon). This mediated response to the flow promotes efficient circulation and, in turn, optimizes gas exchanges. These flow-affecting RBC properties relate mainly to cell deformability (Depond et al.; McMahon; Semenov et al.; Lee et al.), their adherence to wall endothelial cells (ECs) (McMahon) and self-aggregation (Lazari et al.; Lee et al.).

RBC deformability refers to the ability of the cells to modify their shape to the dynamically changing flow conditions to minimize their resistance to flow and to enable their passage through small blood vessels (Jani et al.). RBC deformability has been postulated to be a significant determinant of RBCs survival (Cranston et al., 1984; Safeukui et al., 2018). In general, during cell aging (*in-vivo* and *in-vitro*) or under different pathological conditions, RBC deformability can be impaired. This phenomenon is induced by modification of cell membrane or cytosol composition and membrane structure, for instance, by the alteration of the scaffolding interactions between membrane and cytoskeleton, and the ion or water contents of RBCs (Badens and Guizouarn, 2016; Huisjes et al., 2018). This reduction of RBC deformability, may impair RBC passage and lead to splenic (Da Costa et al., 2013; Pivkin et al., 2016) and liver (Matot et al., 2013) sequestration and destruction.

RBC adherence to EC of the blood vessel walls has been considered in recent years to be a prominent catalyst of blood vessel occlusion, particularly in the microcirculation (Yedgar et al., 2008). Under healthy conditions, RBC adherence to ECs is insignificant. However, in various disorders, alterations in the RBC membrane make them adherent to EC (Montes et al., 2002; Wagner et al., 2006; Yedgar et al., 2008; Kucukal et al., 2018), and staked RBCs may block capillaries. This phenomenon has been implicated in the pathophysiology of sickle cell anemia, cerebral malaria, and thalassemia.

RBCs in the plasma aggregate to form rouleaux and rouleaux networks (Lazari et al.; Lee et al.) and the increased concentrations of acute-phase proteins, particularly fibrinogen, results in enhanced erythrocyte aggregation (Ben-Ami et al., 2003).

In addition to their own (specific) role in the blood circulation, these features also affect each other. Thus, McMahon discusses the link between RBC adhesion and deformability. While Lazari et al. analyze the relationship between RBC deformability and aggregation. Research in hemorheology is diverse, interdisciplinary, and uses both experimental and numerical methods. The articles presented in this Research Topic illustrate the diversity of these aspects.

## Oxidative Stress Induces Impairment of RBC Flow-Affecting Properties

Over their lifespan, erythrocytes are regularly exposed to the oxidative stress (OS) and undergo various oxidative damages due to the high oxygen tension in arterial blood and their abundant heme iron content, etc. (Mohanty et al., 2014). Moreover, excessive production of reactive oxygen species (ROS) has been described in sickle cell anemia, thalassemia, malaria, and diabetes (Chirico and Pialoux, 2012; Kavishe et al., 2017; Almizraq et al., 2018). In turn, OS contributes to impairment of RBC flow-affecting properties (Yaribeygi et al., 2020), and as illustrated by Balushi et al., treatment with antioxidants can repair these features.

## Flow-Affecting RBC Properties in Health and Diseases

Impairment of the flow-affecting properties of human erythrocyte was associated with the pathophysiology of various diseases, such as sepsis, thalassemia, cerebral malaria, stroke, sickle cell anemia, and diabetes. This relationship has been substantiated by *in-vivo* and *ex-vivo* studies (Lee et al.).

Under normal conditions, RBC deformability allows individual cells to traverse nutritive capillaries. Decreased deformability will result in impaired perfusion and gas exchange in peripheral tissues. Hence, RBC deformability can be regarded as a potential index to diagnose specific diseases (Guo et al., 2016; Lim et al., 2018), for example, not only diabetic kidney disease but also other diabetes-related complications (Lee et al.).

## Methods for Characterization of RBCs Flow-Affecting Properties

Most of the relevant studies have been provided by under *in-vitro* conditions (Ben-Ami et al., 2003; Relevy et al., 2008; Barshtein et al., 2018; Lee et al., 2018; Zaninoni et al., 2018; Zhu et al., 2020), but Jani et al. estimate the erythrocyte mechanical properties *in-vivo* during capillary plug flow. To characterize the mechanical properties of red blood cells, the authors (Jani et al.) combined the capabilities of intravascular microscopy with numerical simulation. Spectrum of *in-vitro* techniques that can be used for characterization of RBCs flow-affecting properties is very broad. This list includes laser ektacytometry (Semenov et al.; Lee et al.), laser tweezers (Lee et al., 2018; Zhu et al., 2020), atomic force microscopy (Steffen et al., 2013), microfluidics (Ben-Ami et al., 2003; Relevy et al., 2008; Guo et al., 2016; Barshtein et al., 2018; Lee et al., 2018; Lim et al., 2018). In this Research Topic, Depond et al. discuss the usefulness of these methods for the characterization of the RBC deformability for malaria patients.

Thus, we can conclude that the features discussed above can act independently and synergistically, affecting blood circulation, and thus, their deterioration can be considered as a powerful catalyst for circulatory disorders.

## Author Contributions

All authors listed have made a substantial, direct and intellectual contribution to the work, and approved it for publication.

## Conflict of Interest

The authors declare that the research was conducted in the absence of any commercial or financial relationships that could be construed as a potential conflict of interest.
